# Validity and Reliability of the Dental Neglect Scale among Romanian Adults

**DOI:** 10.3390/jpm12071035

**Published:** 2022-06-24

**Authors:** Beatrice Adriana Balgiu, Ruxandra Sfeatcu, Christina Mihai, Roxana Romanița Ilici, Ioanina Parlatescu, Laura Tribus

**Affiliations:** 1 Department of Career and Educational Training, University Politehnica of Bucharest, 313 Splaiul Independenţei, 060042 Bucharest, Romania; beatrice.balgiu@upb.ro; 2 Department of Oral Health and Community Dentistry, Faculty of Dental Medicine, Carol Davila University of Medicine and Pharmacy, 17–21 Calea Plevnei Street, 010221 Bucharest, Romania; 3 Department of Preventive Dentistry, Faculty of Dental Medicine, Carol Davila University of Medicine and Pharmacy, 17–21 Calea Plevnei Street, 010221 Bucharest, Romania; christina.mihai@umfcd.ro; 4 Department of Prosthesis Technology and Dental Materials, Faculty of Dental Medicine, Carol Davila University of Medicine and Pharmacy, 17–21 Calea Plevnei Street, Sector 1, 010221 Bucharest, Romania; 5 Department of Oral Pathology, Faculty of Dental Medicine, Carol Davila University of Medicine and Pharmacy, 17–21, Calea Plevnei Street, 020021 Bucharest, Romania; ioanina.parlatescu@umfcd.ro; 6 Department of Internal Medicine, Faculty of Dental Medicine, Carol Davila University of Medicine and Pharmacy, 17–21, Calea Plevnei Street, 020021 Bucharest, Romania; laura.tribus@umfcd.ro

**Keywords:** dental neglect, validation, Romanian adults

## Abstract

The Dental Neglect Scale (DNS) is a well-known measure for assessing behaviours and attitudes related to oral health. However, the factor inconsistency revealed by the literature involves further investigations. The study focuses on the validation of the DNS in the case of a sample of the adult population from Romania. In this regard, data were collected online from 872 adults (616 females and 256 males). DNS reliability was examined from the perspective of internal consistency. Convergent validity was performed by associating DNS with different scales from the oral health field. In order to examine and confirm the factorial structure, the sample was broken down into two subsamples which made the subject of the exploratory factorial analysis (EFA) and confirmatory factorial analysis (CFA), respectively. DNS-RO is positively associated with the scale which measures the values related to oral health (OHVS) and negatively with those that assess the impact of the oral health on life quality (OHIP-14), the distrust of the benefits of oral health services (R-DBS), and reduced need for oral care (DIS). The Cronbach’s α = 0.70, McDonald’s ω = 0.70 and CR = 0.77 are acceptable. Both EFA and CFA (χ^2^/df = 1.13; CFI = 0.99; RMSEA = 0.017; SRMR = 0.059) support the unifactorial structure of the scale. The gender differences show that females evince greater care for oral health than male subjects. The study shows that the DNS-RO can be used to assess the behaviours and attitudes towards oral health in the case of the Romanian adult population in epidemiological studies and health promotion programs through health education.

## 1. Introduction

The concept of dental neglect is defined as the failure to maintain oral health and the neglect of one’s dental hygiene [1]. The characteristics of dental neglect have to do with the failure to provide oral care (hygiene, adequate food, professional service), the inability to seek treatment for dental problems [2], and the absence of values for oral health [3]. All these aspects are to be found in the Dental Neglect Scale (DNS), one of the essential instruments for the examination of dental health care versus dental health neglect. The scale assesses the extent to which individuals take care of their dental health, go to the dentist, and value dental care [4,5].

## 2. Dental Neglect Scale

DNS was translated and validated in various cultural contexts, and the psychometric properties of the scale were tested on samples of adults, with good results [6,7], teenagers [8,9], children [10,11,12], and students from various domains of study [13,14].

However, there are two aspects that have not been clarified in the case of DNS. Firstly, the fact that research highlighted the inconsistency regarding the factorial structure of the scale. Thus, studies identify two factors by means of EFA, in the case of teenagers [8], and of emerging adults [4]; this is similar to the first version of DNS, the 7-item version made for children [1]. In the case of the samples of Norwegian adults, there is only one factor that accounts for 37% of the variance of the scale [6]. Thus, this research created the need for the subsequent psychometric assessment of DNS. Another possible limitation in the studies regarding DNS consists in the fact that the psychometric properties of the instrument were much less examined in the case of adults than in the case of children [10,12].

Initially, DNS was developed and validated on Australian children with the purpose of assessing the latter’s dental neglect by using their parents’ answers [1]. Subsequently, the scale was adapted for adults by retaining six out of seven items established for the children’s version [4,5].

Studies have demonstrated the association of high scores of DNS with the severity of tooth decay, missing teeth [14,15,16], few visits to the dentist [8,14,17], and the unsatisfactory assessment regarding one’s own dental health [6]. The average scores of the scale differ depending on how educated the population is: from 10.18 in the case of Indian teenagers [9] and 13.20 ± 3.80 in the case of American teenagers [8] to 15.61 ± 2.40 in the case of educated young Nepalese population [18] and 19.71 ± 3.94 in the case of young educated Indians [13]. The Cronbach coefficients obtained in various samples showed acceptable internal consistency: 0.71 [5], 0.60 [8], 0.65 and 0.57 [6].

The DNS scores were negatively associated with instruments that measure the impact of dental health on quality of life (Oral Health Impact Profile-14) [19,20], reactions regarding dental procedures and services (Revised-Dental Beliefs Survey), dental fear (Dental Fear Survey) [20,21]. DNS had positive relations with the scales that value oral health (Oral Health Values Scale) [20,22]. The literature also demonstrates [5] that DNS is related to scales that measure unhealthy behaviors of addressability to the dentist (Dental Indifference Scale–DIS): both DNS and DIS are associated with self-reported oral health and oral health impact characteristics, but the two scales measure different constructs.

The relation between dental neglect and the impact of dental issues on the quality of life proved to be significant since people with high dental neglect also have low life quality; in addition, self-reported dental health is considered to be weak in the case of the individuals who neglect their dental health [4,5,15,18,23].

As for gender differences, certain studies do not find any differences in the diagnosis made with DNS [8,15], while other pieces of research identify high scores of dental neglect in the case of male subjects who reported that they have much dental plaque and tooth decay lesions and that they use dental services only when they have a health issue [4].

Given mentioned above and the practical utility of DNS in the field of epidemiology and preventive dentistry in relation to the promotion of oral health through education [4,5,6], as well as the absence of some valid instruments of the DNS type for the evaluation of the Romanian population, we proposed to adapt DNS (DNS-RO) in the case of a sample of Romanian adults.

## 3. Materials and Methods

### 3.1. Translating and Adapting the Scale

During the process of the translation of the scale, the authors followed the recommendations established by WHO [24]. The first stage consisted in translating the scale from English into Romanian by two bilingual Romanian dentists. The comparison and the synthesis of the two translations made by the authors of the study led to the Romanian version of the scale. In the second stage, two other dentists (fluent English speakers) made the blind translation of this last version of the scale. The differences found upon comparing the translations were revised until the translators agreed on the last version. The last stage consisted in deciding on the final version of the scale. The latter was pretested with regard to the format, the clarity, and the difficulty of understanding the items and the instructions on a sample of 35 adults, native in Romanian, in the 24 to 30 age bracket. The respective pre-test did not produce significant changes in the structure of the scale.

### 3.2. Ethical Consideration

The study has been conducted in full accordance with ethical principles, including the 1975 World Medical Association Declaration of Helsinki, as revised in 2013. The study was approved by the Ethical Commission of the “Carol Davila” University of Medicine and Pharmacy, Bucharest (Protocol No. 28447/18.10.2021).

### 3.3. Design and Data Collection

This is a cross-sectional study that included 872 participants (Mean _age_ = 32.98; S.D. = 14.09) who completed an online survey between October and November 2021. A convenience sampling strategy was used. The survey was shared on email campaigns and social media in order to recruit volunteer participants. The eligibility conditions included being over 18 and having residency in Romania. The scale validation is part of a larger project which contains a questionnaire set that assesses behaviours, attitudes, and the perception of the adults’ dental health. The total time for the completion of answers was approximately 10–12 min. The questionnaire was secured to make sure that a participant is allowed to complete it only once. An introductory text posted above the set of instruments informed the participants with regard to the object of research, the whole procedure, and the informed consent. The participation was anonymous in order to control the effect of social desirability. There were no financial rewards. From the very beginning, we underlined the fact that the respondent could stop filling in the questionnaire whenever s/he felt like it with no consequences.

### 3.4. Measures

**The Dental neglect scale–DNS** [5] is used to measure the adults’ behaviours and attitudes involved in the care versus the neglect of dental health. The six items out of which one is reverse are assessed from 1*–Definitely No* to 5–*Definitely Yes* [8]. Sample item: *I keep up my home dental care.* Reduced scores show a greater neglect of dental health. The total score is obtained by adding up the score of all items, considering the reverse item, and it varies between 6 and 30 [5,8].

**Dental beliefs survey-R–R-DBS** [25] measures the patients’ attitudes and beliefs with regard to their relationship with the dentist and dental services. The instrument includes the following subscales: Professionalism, Comfort, Communication, and Implication. The 28-item version, assessed on a 5-item scale (ranging from 1–*never* to 5–*nearly always*) is adapted and validated on a group of Romanian adults [22]. All statements are positive and the score of the items for each factor is calculated. In the case of the present study, there is good reliability: for the total score, the coefficients are the following: α = 0.96 (95% CI—0.94–0.97), ω = 0.96 (95% CI—0.95–0.97), and CFA shows acceptable coefficients: χ^2^/df = 4.08; CFI = 0.94; RMSEA = 0.060; SRMR = 0.037.

**Oral health impact profile–OHIP-14** [26,27] assesses the individuals’ perception of the impact of dental issues on one’s wellbeing. OHIP contains 14 items (assessed from 0–*never* to 4–*very often*) distributed on seven subscales with 2 items: Functional limitation, Physical pain, Psychological discomfort, Physical disability, Psychological disability, Social disability, and Handicap. High levels show greater levels of the impact of dental health on life quality. The scale was validated on samples of Romanian adults, and it demonstrated good reliability [28]. For this study, the total score was calculated by using the additive method. The coefficients of internal consistency for the total score are α = 0.93 (95% CI—0.92–0.93), ω = 0.93 (95% CI—0.92–0.94), while CFA shows the following coefficients: χ^2^/df = 4.90; CFI = 0.96; RMSEA = 0.070; SRMR = 0.038.

**Oral health values scale–OHVS** [20] measures the extent to which the individual invests in dental health, and it is made of 12 items assessed from *1–strongly disagree to 5–strongly agree* (out of which 6 are reverse) distributed in 4 subscales: Professional dental care, Appearance and health, Flossing, and Retention of natural teeth. The total score on the scale is between 12 and 60. The scale validated on the general Romanian population showed good psychometric properties [29]. In this study, the internal consistency coefficients for the total score of OHVS are α = 0.76 (95% CI—0.73–0.78) and ω = 0.76 (95% CI—0.73–0.79), while CFA shows the following coefficients: χ^2^/df = 2.43; CFI = 0.96; RMSEA = 0.041; SRMR = 0.034.

**Dental indifference scale–DIS** [17] measures through its 8 items the behavior of addressability to the dentist (the need for dental treatment in different circumstances) and personal oral hygiene. The total score is between 0 and 8. For the present study, the scale was translated from English into Romanian by forward-backward translation, following the recommendations given by WHO in 2020 [24]. Previous studies find Cronbach’s α coefficients between 0.35 [6] and 0.71 [17]. In the current study, the scale has relatively poor internal consistency: α = 0.39 (95% CI—0.32–0.46) and ω = 0.42 (95% CI—0.34–0.48). The factorial structure highlighted the one-dimensionality of the scale. Thus, the CFA shows: χ^2^/df = 1.22; CFI = 0.95; RMSEA = 0.019; SRMR = 0.026.

### 3.5. Sociodemographic Variables

The sociodemographic data we collected are related to the following aspects: gender, age, studies (primary, secondary, university, and post-university), the residence (urban vs. rural), work sector (public, private, others), and the geographic region (the main eight regions of the country were included).

### 3.6. The Hypotheses

**Hypothesis** **1.** Starting from the mentioned literature, we assume that DNS will have a unifactorial structure, given the results reported with regard to large adult samples [6].

**Hypothesis** **2.** We suppose that DNS will negatively correlate with the tests which assess concepts related to dental health, such as life quality in dental health [5,15,20,23] and distrust of dental services and of dentists [5,20,23], and reduced need for addressability to the dentist [17,30]. At the same time, DNS will correlate positively with the scale which measures the values in dental health [20,21,29].

**Hypothesis** **3.** Given the gender differences identified by prior research done with DNS [4,15], we consider that the same pattern is to be found in the case of the present sample.

### 3.7. Data Analysis

The statistical strategies consisted of descriptive analyses (means, standard deviation, and the normality condition of data was verified by calculating skewness and kurtosis). Reliability was examined with the help of α Cronbach and ω McDonald, considered good when ≥0.70 [31,32]. The convergent validity was performed by associating DNS with other four instruments in the domain of oral health: R-DBS, OHIP-14, OHVS, and DIS. The construct validity of the scale was tested with exploratory factor analysis (EFA) and confirmatory factor analysis (CFA). For these types of analyses, the general sample was divided randomly into two subsamples: the first subsample was necessary for the examination of the factorial structure by means of EFA. The latter was made of people in the 18 and 74 age bracket (Mean _age_ = 33.83; S.D. = 14.23) made of 131 males and 305 females. The second subsample was necessary for the verification of the factorial structure by means of CFA and it was made of subjects in the 18 and 74 age bracket (Mean _age_ = 28.68; S.D. = 13.12) out of which 125 males and 311 females.

EFA was based on two essential conditions: the value over 0.80 of the Kayser-Meyer-Olkin coefficient (KMO) [33] and the significance of the sphericity test [34]. In the case of CFA, in order to determine the adequacy degree of the model, the following coefficients were used: χ^2^ (chi-square), df (degrees of freedom), χ^2^/df (criterion chi squared/df), CFI (comparative fit index), TLI (Tucker-Lewis index), RMSEA (root mean squared error of approximation), SRMR (standardized root mean square residual), NFI (Bentler-Bonett normed fit index). The authors considered the following common recommendations: χ^2^/df has an acceptable value if it is <3 [35]. CFI, NFI, and TLI, values close to 0.90 or greater are acceptable to good [36]. RMSEA and its 90%CI and SRMR have good values when they are close to 0.06 [34]. For the gender difference, the Bayes Independent Mann Whitney–Inference procedure has been applied. Differences in age groups, level of education, and work sector in which more groups of subjects are included were calculated with the Kruskal-Wallis and Mann-Whitney tests. All the data were analysed by means of SPSSv22 (IBM, New York, NY, USA) and JASP 0.16.10 (University of Amsterdam, Amsterdam, The Netherlands) programs.

## 4. Results

### 4.1. Socio-Demographic Characteristics

The resulted sample consisted of 872 participants (Mean _age_ = 32.98; S.D. = 14.09; the youngest respondent–18 years, and the older–74 years). The sample consists in 616 female subjects (70.64%; Mean _age_ = 31.17; S.D. = 13.94) and 256 male subjects (29.36%; Mean _age_ = 30.01; S.D. = 14.47). As for studies, most participants have a university diploma (46%), followed by people with high school studies (23.1%) and post-university studies (21.6%). The smallest sample is that of the people with elementary studies (9.3%). Most of the respondents come from the urban environment (83.7%). As for the work sector, 31% work in the public sector, 40% in the private sector, and 29% are not employed. As for the geographical regions, most respondents come from the center and the South-East of Romania (55.8%), and a very small number come from the North-West part of the country (4%).

### 4.2. The Descriptive Analysis

Table 1 shows the average values, the standard deviations, the skewness and kurtosis indicators, and Cronbach’s α if the item is deleted for the six items of the scale. For items one, four, and six the Kurtosis values are over 3 [37] which shows that data do not have a normal distribution for all items. Cronbach’s α = 0.70 (0.67–0.73) and McDonald’s ω = 0.70 (0.66–0.72) show a good internal consistency. The total average score of DNS-RO (gross scores) is M = 25.29 (S.D. = 3.59). The comparison with the average values of other studies, in which they vary between 10.18 and 19.71 [9,13,18], lead us to believe there is a relatively high level of dental care in the case of the analysed sample. The items with the highest average value are *I consider my dental health to be important* (M = 4.78; S.D. = 0.54) and *I keep up my home dental care* (M = 4.72; S.D. = 0.56). The item with the lowest average value: *I control snacking between meals as well as I should* (M = 3.16; 1.28). In addition, percentiles were provided for the total score (Table 2). Corrected item-total correlations show that all values range between 0.35 and 0.58. Considering De Vaus (2002) [38], the values over 0.30 are satisfactory.

The minimum DNS score was 9, while the maximum score was 30. Scale scores were classified into five categories: highly favorable, favorable, neutral, unfavorable, and highly unfavorable attitudes and behaviours.

### 4.3. The Convergent Validity

DNS achieves significant negative correlations of weak level (r = −0.11 and −0.13, respectively, with OHIP-14 and R-DBS and moderate with DIS (r = −0.42) (all at *p* < 0.001) (Table 3).

Therefore, the bigger the neglect of oral health, the bigger the impact on the quality of life (from the perspective of oral health). In addition, high distrust of dental services is linked to the neglect of oral health. At the same time, it is natural for high oral care scores to be inversely related to attitudes of dental indifference. The correlation between DNS and OHVS is positive (r = 0.37; *p* < 0.001), which confirms that the individuals for whom oral health is an important part of general health are the people who take care of their oral cavity.

### 4.4. Gender Differences

The effect of gender on DNS scores was calculated by means of the Bayesian Independent-Sample Inference procedure which is highly precise and powerful [39]. Given the variables are not normally distributed, we used the Mann-Whitney U non-parametric test (No. sample = 2000) [40,41]. The result shows that the total score of DNS is significantly differentiated for the male subsamples (M = 23.23; S.D. = 2.98; SE = 0.18; 95% Credible interval−22.86–23.60) and for the female subsamples (M = 24.11; S.D. = 2.48; SE = 0.10; 95% Credible interval–23.91–24.30) (BF-0 = 24.30; W = 91436.00, R^ = 1.04), which confirms that female subjects take more care of their oral health.

### 4.5. DNS and Other Socio-Demographic Characteristics

In the next stage of the analysis, the average DNS scores and other socio-demographic characteristics (age, education, and work sector) were examined (Table 4). As the data are not normally distributed, the Kruskal-Wallis H test was used, which is based on the hierarchy of data, and Mann-Whitney U was applied to identify significant differences.

There is a significant difference between age groups, mean rank of DNS increasing from one age stage to another (all *p* < 0.001).

Differences were also found depending on the level of education. People with post-graduate education score significantly higher compared to the group with secondary education (z = −4.00) and university (z = −2.81) (*p* < 0.001). Also, there are differences between the respondents with university studies and those with secondary education in favor of the high score for the former (z = −2.01) There is also a moderate difference between people working in the public sphere compared to those coming from the private sphere (z = −2.17) (*p* = 0.03).

### 4.6. The Exploratory Factor Analysis

As shown, the general sample was subdivided randomly into two subsamples which are approximately balanced in terms of gender and age using syntax from SPSS: Data/Select cases/Random sample of case. For subsample 1, for which EFA was carried out, the conditions for the implementation of EFA showed the possibility of applying the latter.

Thus, KMO = 0.78 and Barttlett’s test of sphericity is significantly χ^2^ = 555.8838, df = 15, *p* = 0.000, which thus supports the factoriability of the correlation matrix [33]. The factorial solution (the principal component analysis with varimax rotation) showed the presence of one single factor which accounts for 46.32% of the total variance of the scale. In this case, the coefficients which assess reliability show a good internal consistency (Cronbach’s α and McDonald’s ω < 0.70) (Table 5).

### 4.7. The Confirmatory Factor Analysis

The one-factor model was tested on subsample 2. For the assessment in CFA we used the robust version Diagonally weighted least squares (DWLS) recommended for the data that deviate from normal distribution [42]. Since the critical ratio (c.r.) is 34.45, and the Mardia coefficient is 30.06, the sample can be considerate multivariate non-normal. A robust approach of the management of non-normality in the modelling of the structural equation is bootstrap re-sampling [43,44]. Consequently, we applied bootstrapping with 2000 resamplings (95% confidence interval) in order to solve the non-normality. The fit coefficients we obtained are excellent: χ^2^ = 10.199; df = 9; χ^2^/df = 1.13; CFI = 0.99; NFI = 0.97; TLI = 0.99; RMSEA = 0.017 (90%Confidence Interval-CI 90%–0.000–0.056); SRMR = 0.059; *p* = 0.335 (Table 6).

**Figure 1:**
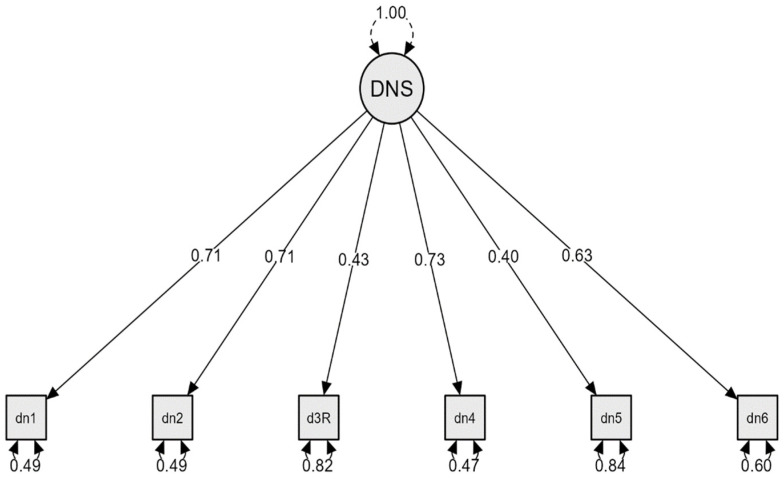
Single-factor model of the DNS with the standardised factor loading. Note: dn1–dn6 = items DNS.

Given the values of the statistical coefficients, we can conclude that the one-factor and 6-item model is a valid one. The factor loading varies between 0.40 and 0.72. Thus, the factor loading are item 1 = 0.71, item 2 = 0.71, item 3 = 0.43, item 4 = 0.73, item 5 = 0.40, and item 6 = 0.63 (Figure 1). The consistency coefficients are acceptable in the case of this subsample, as well. Thus, Cronbach α = 0.71 (0.66–0.74), McDonald ω = 0.69 (0.65–0.73), while composite reliability calculated on the basis of of factor loading (λ) and standard error (ε) is 0.77.

## 5. Discussion

Starting from the fact that a valid instrument of diagnosis of oral self-care is needed in clinical and research examinations, the purpose of the present study is to confirm the psychometric properties of the Romanian version of DNS (DNS-RO).

Research shows that the psychometric properties of DNS-RO are good. The internal consistency is acceptable both in the case of the general sample (Cronbach’α = 0.70 and McDonald’ω = 0.70), and in the case of the two subsamples on which the exploratory and confirmatory analyses were carried out.

Most of the studies analysed the psychometric properties of DNS in association with the status of health and oral hygiene [8,13,45], the number of visits to the dentist [8], or the existence of tooth decay [13]. The number of studies that analysed the convergent validity of DNS by associating it with other validated instruments is underrepresented. In this study, DNS is significantly negatively associated with scales that measure the attitudes of individuals towards dental services (R-DBS), the influence of oral health on life quality (OHIP-14), and the need for dental treatment (DIS), and positively associated with the scale that measures the values in oral health (OHVS). Therefore, as we hypothesized, there is a relation between the neglect of oral health, on the one hand, and distrust in dental services, on the other hand, reduced psycho-social functioning caused by poor oral health and lack of care for one’s own oral health. This result corroborates those studies which demonstrated that individuals who neglect their oral cavity have a low quality of life [4,5,15,23,46], are less oriented towards dental services [5,23], and they do not give any importance to oral health [20,47].

The analysis of the factorial structure carried out by means of the exploratory factorial analysis and verified by means of the confirmatory analysis [48] led to consistent findings regarding the dimensionality of the scale. Although certain research carried out on teenagers concludes that DNS contains two factors [8], namely dental neglect and dental avoidance, the latter are not demonstrated in all the examinations of DNS [6]. The study corroborates the established hypothesis and confirms the six-item unifactorial structure of DNS. Therefore, the results are similar to the unidimensionality of the scale [6].

As for the average value for the total score of DNS-RO, the study shows an average of 25.29, which means much self-oral care within the sample. Given that the sample contains more than 40% of people with university and post-university diplomas, the association between oral care and the status and individual education [5,13,15]. The gender differences confirm studies that highlight that female subjects have higher scores when it comes to dental health in comparison with male subjects [4,15]. The respective result is demonstrated in the present study which shows that female subjects take more care about their oral health than male subjects. In fact, the data based on gender show that women are more oriented towards dental services [49] and have positive attitudes towards dental hygiene [50]. Another similar result with studies is the differentiation according to the level of education [9,13,18]. In this sample, it is verified that as the respondents have a high level of education, they also have high attitudes and behaviors regarding oral health.

*Limitations*: The limits of the research are related to demographic data. One of them consists in the lack of balance related to age (more than 50% of respondents are young), therefore we need to be cautious when generalizing the results to other populations. Another aspect related to the sample is the lack of gender balance. The presence of a larger number of women and those with higher education in the sample is explained by the fact that they are more receptive to completing online surveys. Despite these limitations, the results support the fact that DNS-RO is a promising instrument that can be used in the assessment of the behaviours related to oral care.

## 6. Conclusions

The study is the first to look into the adaptation of DNS within the Romanian general population. The findings we obtained lead us to believe that DNS-RO can be considered a new valid instrument for the assessment of the attitudes and behaviours regarding oral health, as it is easy to apply and to score. The implications for research and practice stem from the fact that it offers clinicians and researchers a valid instrument regarding the assessment of behaviours related to dental care and the identification of educational needs. Thus, dental issues would be better understood, and one could plan the promotion of health better. Thus, the identification of preventive healthcare priorities would be improved, and health promotion could be adequately planned along with clinical indicators, by identifying the need for health education.

## Figures and Tables

**Table 1:** Descriptive statistics of all items of the DNS-RO (N = 872).

Items	M	S.D.	Skew.	Kurt.	α If Item Deleted	Corrected Item-Total Correlation
1. I keep up my home dental care	4.72	0.56	−2.48	8.07	0.64	0.59
2. I receive the dental care I should	4.33	0.95	−1.34	1.07	0.61	0.57
3. I need dental care, but I put it off (reversed)	3,71	1.31	−0.68	−0.77	0.69	0.38
4. I brush as well as I should	4.56	0.69	−1.79	3.83	0.64	0.54
5. I control snacking between meals as well as I should	3.16	1.28	−0.09	−0.95	0.70	0.35
6. I consider my dental health to be important	4.78	0.54	−2.92	9.79	0.66	0.50
ω = 0.70 (0.67–0.73)						
α = 0.70 (0.66–0.72)						

Note: M–Mean; S.D.–standard deviation; Skew-Skewness; Kurt-Kurtosis.

**Table 2:** Percentiles for the DNS.

Percentiles	Scores	Qualitative Interpretation (Attitudes towards Oral Health Care)
10th	20.00	Highly unfavorable
25th	23.00	Favorable
50th	26.00	Neutral
75th	28.00	Unfavorable
90th	30.00	Highly favorable

**Table 3:** Intercorrelations between DNS and other measures.

Measures	1	2	3	4
1. DNS	-			
2. OHIP-14	–0.11 ***	-		
3. R-DBS	–0.13 ***	−0.20 ***	-	
4. OHVS	0.37 ***	–0.16 ***	–0.33 ***	-
5. DIS	–0.42 ***	0.12 ***	0.25 ***	–0.49 ***

*** *p* < 0.001.

**Table 4:** Mean DN score and S.D. by age, level of education and work sector.

Groups	Mean Rank	Chi-Square (H)
Age group		
18–29 years	379.27	88,743 df = 2; *p* < 0.001
30–49 years	508.92	
50+ years	595.36	
Education *		
High school	389.03	16,433 df = 2; *p* < 0.001
Universitary	431.71	
Post-universitary	490.44	
Work sector		
Public	213.76	4.73 df = 1; *p* = 0.030
Private	188.61	

* the respondents with primary education, which represents a small percentage of the entire sample, were eliminated.

**Table 5:** The result of the exploratory factor analysis.

Items	Component 1
Item 1	0.80
Item 2	0.72
Item 3	0.60
Item 4	0.70
Item 5	0.54
Item 6	0.68
Variance %	46.32%
Cronbach’s α	0.70 (0.65–0.74)
McDonald’s ω	0.70 (0.65–0.74)

**Table 6:** Goodness-of-fit indices of the confirmatory factor model.

Models	χ^2^	df	χ^2^/df	CFI	NFI	TLI	RMSEA (_90%_CI)	SRMR	*p*
Default model	10.199	9	1.13	0.99	0.97	0.99	0.017 (0.000–0.056)	0.059	0.335

## Data Availability

The data presented in this study are available from the corresponding authors upon reasonable request.
